# Pancreatic cancer: The RAS paradigm shift

**DOI:** 10.1371/journal.pmed.1005283

**Published:** 2026-10-29

**Authors:** Samuel P. Soff, Eileen M. O’Reilly

**Affiliations:** 1 Department of Medicine, Weill Cornell Medicine, New York, New York, United States of America; 2 Department of Medicine, Memorial Sloan Kettering Cancer Center, New York, New York, United States of America

## Abstract

Treatment approaches for pancreatic adenocarcinoma, an aggressive disease with poor prognosis, are evolving beyond cytotoxic chemotherapy. In this Perspective, Samuel Soff and Eileen O’Reilly discuss recent breakthroughs and clinical trial results of RAS-targeting small molecule inhibitors, as well as the challenges of treatment resistance.

## Teaser

Treatment approaches for pancreatic adenocarcinoma, an aggressive disease with poor prognosis, are evolving beyond cytotoxic chemotherapy. Recent advances in RAS targeting with small molecules are redefining therapeutic paradigms.

## Introduction

Pancreatic ductal adenocarcinoma (PDAC) is one of the most lethal malignancies, with a 5-year survival rate of approximately 13%, among the lowest of all cancers [1]. PDAC typically causes nonspecific symptoms, and most patients present with advanced disease, typically with liver, lymph node, lung, and peritoneal metastases [1]. Existing front-line therapeutics for metastatic disease include modified FOLFIRINOX (a four-drug chemotherapy regimen), NALIRIFOX (a FOLFIRINOX variant with liposomal irinotecan), or gemcitabine and nab-paclitaxel (GnP); however, median survival remains <1 year with these treatment regimens [1]. In addition to modest survival benefits, these regimens incur significant toxicity, limiting their utility in frail/older patients [1,2]. Therefore, there remains an urgent need for more effective, less toxic treatment options, moving beyond cytotoxic therapy.

Targeted therapies have an established role in a small number of genomically defined settings in PDAC, including platinum agents and poly-ADP-ribose polymerase inhibitors in patients with germline/somatic *BRCA*1/2 or *PALB*2 mutations [1,3]. Pembrolizumab has established use for microsatellite instability-high or deficient DNA mismatch repair tumors, although the application is relevant to only 1%–2% of patients with PDAC [1,3].

## RAS targeting in pancreatic cancer

PDAC is characterized by alterations in the rat sarcoma (*RAS*) mitogen-activated protein (*MAP*) kinase pathway. Normally, the RAS enzyme cycles between an inactive guanosine diphosphate (GDP)-bound “off” state and an active guanosine triphosphate (GTP)-bound “on” state, promoting proliferation and survival when active. Mutations in this oncogene lead to a constitutively “on” state, contributing to the process of tumor proliferation, survival migration and invasion in pancreatic, lung, and colorectal cancer [1,4]. Mutations in the *KRAS* oncogene occur in 90–95% of PDAC, most commonly at codon 12; including G12D (~40%), G12V (~30%), and G12R (~15%), making KRAS a compelling therapeutic target [1,2,5,6].

Until now, RAS was historically considered “undruggable” due to limited drug binding sites and high affinity for GTP [2]. This changed with the targeting of KRAS G12C in PDAC, with sotorasib (KRAS G12C inhibitor) demonstrating an overall response rate (ORR) of 21%, a median progression free survival (PFS) of 4.0 months, and median overall survival (OS) of 6.9 months [2]. Similarly, another G12C inhibitor, adagrasib, demonstrated a moderate survival benefit and response rate (see Table 1). These observations underpin utility for RAS targeting in PDAC, acknowledging the rarity of KRAS G12C in only 1%–2% of PDAC [2,4].

**Table 1:** Clinical trials of KRAS-targeted therapies in pancreatic ductal adenocarcinoma (PDAC).

Target	Trial	Cohort	Phase	Sample Size	Treatment	Control	Outcomes (95% CI)	Notes	Study Status	Reference
KRAS G12C	CodeBreaK 100 (NCT03600883)	Metastatic PDAC with KRAS G12C, ≥1 prior systemic therapy	Phase 1–2	38	Sotorasib (KRAS G12C inhibitor)	None	OR 21% (10%−37%); Median PFS 4.0 months (2.8–5.6); Median OS 6.9 months (5.0–9.1)	Established a targeted therapy for G12C	Published	PMID 36546651
	KRYSTAL-1 (NCT03785249)	Metastatic or unresectable solid tumor with KRAS G12C, no further available standard therapy	Phase 2	21 (PDAC specifically)	Adagrasib (KRAS G12C inhibitor)	None	OR 33.3% (14.6%−57.0%); Median PFS 5.4 months (3.9–8.2); Median OS 8.0 (5.2–11.8)	Established a targeted therapy for G12C	Published	PMID 37099736
KRAS G12D	NCT05382559	Metastatic or unresectable solid tumors with KRAS G12D, ≥1 prior systemic therapy	Phase 1	21 (PDAC specifically, at higher dose 600 mg weekly)	Setidegrasib (KRAS G12D protein degrader)	None	OR 24% (8%−47%); Median PFS 3.0 (1.4–6.9); Median OS 10.3 (4.2–13.0) [among patients with higher dose 600 mg]	Established a targeted therapy for G12D	Published	PMID 41879829
	NCT06520488	Metastatic or unresectable PDAC with KRAS G12D, no prior systemic therapy	Phase 1b-2	30	HRS-4642 (KRAS G12D inhibitor) plus Gemcitabine + Nab-Paclitaxel	None	OR 63.3% (43.9%−80.1%); Median PFS 13.1 months (12.0-not reached); 12 month OS 83.3% (64.5%−92.7%)	Demonstrated efficacy for KRAS G12D inhibition plus chemotherapy	Published	PMID: 42426224
	NCT06179160	Metastatic or unresectable PDAC with KRAS G12D, ≤1 prior systemic therapy	Phase 1	29 (patients with higher dose 1,200 daily)	INCB161734 (G12D inhibitor) +/− standard chemotherapy	None	Partial response 34%; Disease control 86%; Molecular response 72% (95% CI not reported) [among patients who received higher dose 1,200 mg daily]	Investigating a novel type of G12D inhibitor, +/− chemotherapy	Ongoing trial, data are preliminary	NCT06179160
	RASolute 305 NCT07621718	Metastatic or unresectable PDAC with KRAS G12D mutation, no prior systemic therapy	Phase 3	670 (estimated)	Zoldonrasib (G12D inhibitor) + standard chemotherapy	Placebo + standard chemotherapy	N/A	Investigating Zoldonrasib + chemotherapy as first line treatment for G12D mutant	Ongoing trial, no available data	NCT07621718
PanRAS	NCT05379985	Metastatic or unresectable PDAC with RAS mutation, ≥1 prior systemic therapy	Phase 1–2	26 (subgroup with RAS G12 mutation, at higher dose 300 mg daily)	Daraxonrasib (PanRAS inhibitor)	None	OR 35% (17%−56%); Median duration of response 8.2 months (3.8 to not reached); Median PFS 8.5 months (6.7–10.5); Median OS 13.1 months (10.9 to not reached)	Preliminarily (phase 1/2 trial) established Daraxonrasib as safe/ effective second line treatment	Published	PMID 42090791
	RASolute 302 NCT06625320	Metastatic or unresectable PDAC, ≥1 prior systemic therapy	Phase 3	500 [248 treatment + 252 control]	Daraxonrasib (PanRAS inhibitor)	Standard chemotherapy (mFOLFIRINOX or GnP)	Median OS 13.2 months (10.0 to not reached) vs. 6.6 months (5.4–8.2); Mortality HR 0.40 (0.30–0.54); Median PFS 7.3 months (6.3–8.1) vs. 3.5 months (2.9–3.8); OR 33.2% (27.0–39.9) vs. 11.8% (7.8–16.8) (among KRAS G12 population) [all in favor of treatment vs. chemotherapy]	Established Daraxonrasib has significant benefit over chemotherapy for second line treatment of mPDAC; **First phase 3 trial with PanRAS targeted therapy**	Published	PMID 42223072
	NCT06445062 (data from subgroup analysis)	Metastatic or unresectable PDAC with KRAS mutation, no prior systemic therapy	Phase 1–2	1,130 (estimated); 40 in preliminary subgroup analysis	Daraxonrasib/Zoldonrasib with various chemotherapy; prelim data of Daraxonrasib + GnP	None	Subgroup analysis with Daraxonrasib + GnP: ORR 58% (41%−73%); DCR 90% (76%−97%); 6-month PFS 84% (68%−93%)	Comparing combinations of Daraxonrasib and Zoldonrasib with chemotherapy for first line	Ongoing trial, data are preliminary	NCT06445062
	Rasolute 303 NCT07491445	Metastatic or unresectable PDAC, no prior systemic therapy	Phase 3	900 (estimated)	Arm A: Daraxonrasib Arm B: Daraxonrasib + GnP	Standard chemotherapy (mFOLFIRINOX or GnP)	N/A	Investigating Daraxonrasib +/− chemotherapy as first line treatment	Ongoing trial, no available data	NCT07491445
	Rasolute 304 NCT07252232	PDAC with curative intent surgical resection, no recurrence or metastatic disease	Phase 3	500 (estimated)	Daraxonrasib (PanRAS inhibitor)	Standard of care observation	N/A	Investigating Daraxonrasib maintenance after resection and neoadjuvant/adjuvant chemotherapy	Ongoing trial, no available data	NCT07252232
PanRAS + PRMT5i	NCT06922591	Metastatic or unresectable PDAC with MTAP deletion and RAS mutation, ≥1 prior systemic therapy	Phase 1–2	Daraxonrasib: 12 Zoldonrasib: 27	Vopimetostat (PRMT5i) with Daraxonrasib (PanRASi) or Zoldonrasib (KRAS G12Di)	None	Daraxonrasib group: OR 92%; 6 month PFS 90%; DCR 100% Zoldonrasib group: OR 52%; 6 month PFS 74%; 96% DCR (95% CI not reported)	Investigating the utility of PRMT5i with Daraxonrasib in second line treatment	Ongoing trial, data are preliminary	NCT06922591

Daraxonrasib (RMC-6236), a novel tricomplex inhibitor, targets the active GTP-bound (RAS-ON) state across multiple oncogenic RAS and wild-type variants, expanding targeted therapy to nearly every patient with PDAC [5]. The phase 3 RASolute 302 trial randomized five hundred patients with previously treated metastatic PDAC (92% KRAS G12 mutated tumors) to daraxonrasib compared to best standard chemotherapy [5]. In the G12-mutated population, daraxonrasib demonstrated a median OS of 13.2 months versus 6.6 months in the chemotherapy group [5]. PFS was 7.3 months with daraxonrasib versus 3.5 months with chemotherapy. The most common side effects for daraxonrasib were rash, diarrhea, stomatitis, nausea, and vomiting; however, it was generally tolerated more favorably than chemotherapy, with a lower incidence of grade ≥3 adverse events (61.8% versus 69.6%) and treatment-related discontinuation rates (1.2% versus 11.2%) for daraxonrasib versus chemotherapy [5].

These results are without precedent in PDAC and the significance extends beyond PDAC, firmly discrediting the notion that RAS is ‘undruggable’. These results signal an opportunity for nonchemotherapy treatment paradigms for PDAC, and expand eligible populations for treatment consideration, including older/frail patients. Daraxonrasib received regulatory approval by the Food and Drug Administration (FDA) in the US in August 2026 and is under expedited review by global authorities.

Current trials are expanding the role of the tricomplex RAS inhibitors in other settings in PDAC (Table 1). RASolute 303 is a three-arm phase 3 study, evaluating daraxonrasib monotherapy and daraxonrasib + GnP, compared to standard care chemotherapy in treatment-naive patients with metastatic PDAC, and moves RAS targeting to front-line treatment of metastatic PDAC (NCT07491445). RASolute 304 is investigating daraxonrasib compared to observation as an adjuvant option following completion of standard of care surgery and neoadjuvant/adjuvant therapy (NCT07252232).

Other combination approaches include the use of Protein arginine methyltransferase 5 (PRMT5) inhibitors, which disrupt gene splicing, cellular signaling, and specifically target methylthioadenosine phosphorylase-deleted PDAC, present in ~20%–30% of PDAC [6]. In pre-clinical testing, PRMT5i combined with daraxonrasib (pan-RASi), MRTX1133 (G12Di), or adagrasib (G12Ci) led to a significant reduction in cell division and tumor regression, more-so than the single drugs achieved independently [6]. Preliminary data from an ongoing phase 1/2 trial demonstrated that PRMT5i plus daraxonrasib resulted in an encouraging 92% ORR, with 90% 6-month PFS and 100% disease control rate, supporting further investigation with a phase 3 trial (NCT06922591) [7] (Table 1).

## Allele/mutant-specific inhibitors

KRAS G12D is the most prevalent mutation in PDAC, accounting for approximately 40% of cases, and multiple G12D-specific agents are under active investigation [8,9]. Setidegrasib, a G12D protein degrader, demonstrated a 24% objective tumor shrinkage, with a median PFS of 3 months and OS 10.3 months; notably, 67% of patients received setidegrasib as third-line treatment [8]. HRS-4642, another G12D specific inhibitor, combined with GnP, demonstrated a 63.3% tumor shrinkage as first line therapy in metastatic disease [9]. Multiple ongoing trials are investigating additional G12D inhibitors, such as Zoldonrasib (RMC-9805), a tricomplex G12D inhibitor (NCT06445062, NCT07621718), or INCB161734, a selective ON/OFF KRAS G12D inhibitor (NCT06179160), with encouraging preliminary data.

## Beyond pancreatic cancer

As KRAS mutations occur in other cancers, namely non-small-cell lung cancer (NSCLC) and colorectal cancer (CRC), their clinical utility extends beyond PDAC. KRAS G21C inhibitors such as adagrasib, sotorasib, and olomorasib have been applied in both NSCLC and CRC, as has setidegrasib (G12D degrader) for NSCLC [2,4,8,10]. The first generation G12C inhibitors adagrasib and sotorasib have demonstrated similar responses to those in PDAC, with ORR 30%–40%, and PFS of 6–8 months [10]. Second generation inhibitors, such as olomorasib, have demonstrated improved responses, with ORR 45%–55% and PFS exceeding that of first generation agents [10]. Daraxonrasib has shown promise in NSCLC as well, with the phase 1/2 NCT05379985 trial demonstrating a 37% ORR with daraxonrasib in previously treated advanced RAS-mutant NSCLC [11]. Ongoing trials are further investigating the role of pan-RAS inhibitors (NCT06607185) and mutant-specific inhibitors (NCT06252649, NCT07659795, and NCT07222566) in CRC and NSCLC, both as monotherapy and in combination with chemotherapy.

## Treatment resistance

RAS targeted treatment is evidently practice-changing, however, there are challenges to be addressed. Among patients with G12C mutated NSCLC/CRC treated with G12Ci (adagrasib and sotorasib), 45% demonstrated mechanisms of resistance following treatment, with 18% demonstrating multiple coinciding mechanisms [12]. These include additional KRAS alterations, oncogenic fusions and loss-of-function mutations. Similarly, among patients with PDAC treated with daraxonrasib, 59% had treatment emergent genomic alterations in the RAS signaling pathway, as well as alterations in other signaling pathways such as receptor tyrosine kinase (RTK) [13]. To address resistance, combination strategies are under investigation to improve response rates by targeting both de novo and acquired resistance mechanisms. Daraxonrasib, combined with agents targeting DNA damage response, RTKs, or a second RAS G12 inhibitor such as zoldonrasib (G12Di) decreased resistance in pre-clinical models [13]. Combinations with immune checkpoint blockade drugs (e.g., anti-programmed death; anti-PD1 inhibitors) have improved responses and delayed resistance, as KRAS inhibitors can remodel the tumor microenvironment by reducing immunosuppressive cytokines, leading to synergy with PD1/PDL1 inhibitors [10]. These studies may guide potential combination strategies in RAS mutated malignancy for further clinical investigation.

## Conclusions

The treatment of PDAC is undergoing a paradigm shift, from chemotherapy for all to targeted therapy with RAS inhibitors for all, underscored by the recent FDA approval of daraxonrasib. The results and implications of the RASolute 302 trial are profound and immediate, demonstrating RAS is no longer “undruggable”. Next steps include evaluating RAS inhibitors as first line treatments (in neoadjuvant and adjuvant settings), addressing acquired resistance, and identifying optimal combination strategies in PDAC and other RAS-driven malignancies.
